# The Effect of *Bauhinia bowkeri* Extracts on Hypercholesterolemia: Insights from In Vitro and In Silico Investigations

**DOI:** 10.3390/plants14060979

**Published:** 2025-03-20

**Authors:** Siphelele T. Thethwayo, Evelyn Madoroba, Sphamandla Masikane, Andrew R. Opoku, Nkosinathi D. Cele

**Affiliations:** 1 Department of Biochemistry and Microbiology, Faculty of Science, Agriculture, and Engineering, University of Zululand, Private Bag X1001, KwaDlangezwa 3886, South Africa; siphelelethethwayo98@gmail.com (S.T.T.); madorobae@unizulu.ac.za (E.M.); opokua@unizulu.ac.za (A.R.O.); 2 Department of Chemistry, Faculty of Science, Agriculture and Engineering, University of Zululand, Private Bag X1001, KwaDlangezwa 3886, South Africa; masikanes@unizulu.ac.za

**Keywords:** *Bauhinia bowkeri*, HMG-CoA, anti-hypercholesterolemic, molecular docking, GC-MS, antioxidants

## Abstract

Despite the many current cholesterol-lowering drugs on the market, the persistent surge of hypercholesterolemic-related complications ignites a fascinating search for the discovery of novel therapeutics. This study aimed at investigating the anti-hypercholesterolemic effect of *Bauhinia bowkeri* extracts. The plant material was sequentially extracted with n-hexane, dichloromethane (DCM), and 70% ethanol. The phytochemical constituents of the extracts were analyzed through GC-MS and the antioxidant activity of the extracts was screened against a wide range of free radicals (ABTS, DPPH, hydroxyl radical, and nitric oxide). The extracts were also screened for the metal iron chelating and reducing power potential. The enzyme inhibitory activity of the extracts on pancreatic lipase, cholesterol esterase, and HMG-CoA reductase as well as the bile acid binding capacity were evaluated. Among the total of 122 compounds detected in the three extracts, only 7 compounds (E-15-Heptadecenal, Diethyl Phthalate, 9,12,15-Octadecatrienoic acid ethyl ester, (Z,Z) Tetradecane 5-methyl, and Octadecane 5-methyl) were found to be common in all the extracts. The extract displayed a varying degree of efficiency on free radicals with IC_50_ values ranging from 0.07 mg/mL to 0.41 mg/mL. A concentration-dependent inhibition of pancreatic lipase and cholesterol esterase activities, along with a reduction in the bile-binding capacity exhibited by the extracts, was noted. In silico investigations of some of the phytoconstituent revealed significant inhibition of HMG-CoA reductase, cyclooxygenase, and hormone-sensitive lipase with a binding affinity that ranged between −5.1 and −7.0 kcal/mol. These findings suggest that *Bauhinia bowkeri* extracts possess potential antioxidant and anti-hypercholesterolemic properties.

## 1. Introduction

Hypercholesterolemia, a condition characterized by elevated blood cholesterol levels, is a major global health concern and a leading cause of morbidity and mortality [1]. It is a primary risk factor for cardiovascular diseases (CVDs) and their associated complications, including atherosclerosis [2]. According to the World Health Organization (WHO) Global Status Report, non-communicable diseases (NCDs) account for approximately 41 million deaths annually, representing 71% of all global fatalities. Among these, CVDs contribute the most, with an estimated 17.9 million deaths per year [3].

Hypercholesterolemia results from a complex interplay between genetic predisposition and environmental factors, such as dietary habits, sedentary lifestyle, and oxidative stress. Dysregulation of lipid metabolism, particularly the impaired cholesterol homeostasis and abnormal metabolism of cholesterol-rich lipoproteins, plays a critical role in disease progression [4]. Elevated levels of low-density lipoprotein (LDL) cholesterol contribute to cholesterol accumulation in arterial walls, leading to the formation of atherosclerotic plaques. This process compromises vascular integrity and increases the risk of cardiovascular events, including myocardial infarctions and ischemic strokes [5,6].

Pharmaceutical interventions for hypercholesterolemia include 3-hydroxy-3-methylglutaryl-CoA reductase (HMG-CoA) inhibitors (statins), bile acid sequestrants, nicotinic acid, fibrates (e.g., Gemfibrozil), and Ezetimibe for cholesterol absorption [7]. However, these agents are often associated with limitations, including high costs; restricted accessibility, especially in low-resource settings; and adverse effects, such as headaches, dizziness, nausea, nosebleeds, sore throat, constipation, erythema multiforme, acute pancreatitis, and severe contraindications [8]. Notably, recent studies suggest that multi-targeting therapeutic approaches may offer superior disease-modifying efficacy compared to single-target strategies [9]. Consequently, there is growing interest in exploring natural compounds with lipid-lowering properties as alternative therapeutic agents.

*Bauhinia bowkeri*, a member of the Fabaceae family, is native to the valley bushveld regions of Pongola, KwaZulu-Natal, South Africa [10]. Traditional medicine practitioners use various formulations derived from *B. bowkeri* bark to manage a range of ailments. Previous studies have reported the antioxidant [11,12], anti-diabetic [13], anti-inflammatory [14], anti-microbial [15], and anti-Alzheimer’s [16] properties of *B. bowkeri* extracts. Given these bioactivities, this study aimed to further investigate the potential anti-hypercholesterolemic properties of *B. bowkeri* bark extracts using both in vitro and in silico approaches.

## 2. Results

### 2.1. Percentage Yield

The sequentially extracted yields of the crude extracts obtained from the powdered bark material of *B. bowkeri* using n-hexane, DCM, and 70% ethanol solvents were 0.28%, 0.29%, and 2.38% (*w*/*w*), respectively.

### 2.2. Total Phenolic and Flavonoids Content

**Figure 1:**
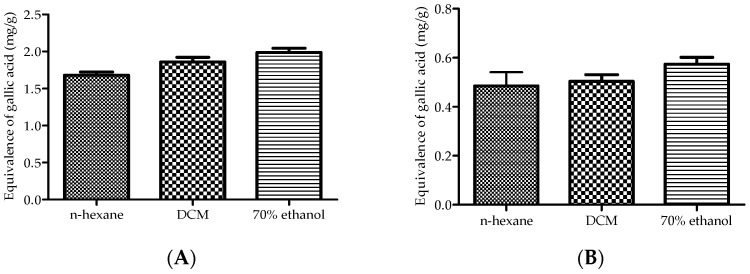
Total phenolic (**A**) and flavonoid (**B**) contents of the plant extracts. The values are expressed as the mean ± standard deviation (SD). A one-way analysis of variance (ANOVA), followed by the Dennett post hoc test, was used to compare the groups, with *p* ≤ 0.05 considered to be statistically significant.

The total phenolic and flavonoid contents of the plant extracts are presented in Figure 1. All the extracts exhibited the presence of both phenolic (A) and flavonoid (B) contents. Notably, the 70% ethanol extract demonstrated the highest phenolic content (1.99 ± 0.10 mg/g) and flavonoid content (0.57 ± 0.05 mg/g), significantly surpassing those of the n-hexane extract (1.68 ± 0.07 mg/g and 0.49 ± 0.01 mg/g, respectively) and dichloromethane (DCM) extract (1.86 ± 0.11 mg/g and 0.50 ± 0.05 mg/g, respectively).

### 2.3. FT IR Analysis

**Figure 2:**
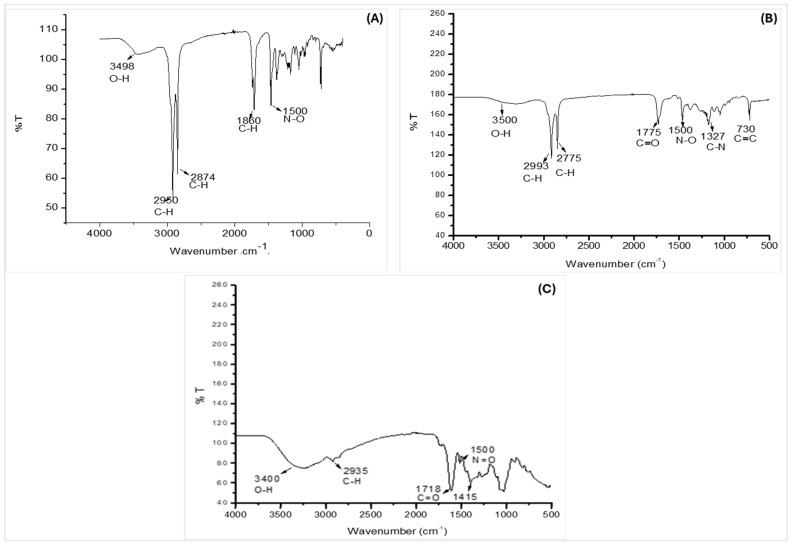
Represents FTIR spectra of *B. bowkeri* extracts obtained with n-hexane (**A**), dichloromethane (DCM) (**B**), and 70% ethanol (**C**), showing the presence of functional groups (n = 3).

The Fourier transform infrared (FTIR) spectroscopy results of the bark extracts from *B. bowkeri* are presented in Figure 2. The spectra of all three sequential extracts (n-hexane, dichloromethane (DCM), and 70% ethanol) exhibited absorption peaks indicative of common functional groups. Notably, hydroxyl (O-H) groups were detected, as evidenced by the absorption peaks in the regions of 3000–3600 cm^−1^ and 3600–4000 cm^−1^, corresponding to O-H stretching vibrations. The presence of these functional groups suggests that the extracts may contain phenolic compounds.

### 2.4. Phytochemical Profiling of B. bowkeri

The phytochemical constituents of *B. bowkeri* crude extract were analyzed using gas chromatography-mass spectrometry (GC-MS) and the results are shown in Table 1. A total of 122 compounds were identified across the extracts, with n-hexane (43/122), DCM (29/122), and 70% ethanol (50/122).

Notably, seven compounds were common to all three extracts: E-15-Heptadecenal, Diethyl Phthalate, Hexadecanoic acid ethyl ester, 9,12,15-Octadecatrienoic acid ethyl ester (Z,Z,Z), Tetradecane-5-methyl, and Octadecane-5-methyl. Among these, Tetradecane-5-methyl and Octadecane-5-methyl were the most abundant.

### 2.5. In Vitro Antioxidant Activity

The antioxidant activities of *B. bowkeri* crude extracts were evaluated using various in vitro assays, and the results are presented in Table 2. All the extracts exhibited concentration-dependent scavenging activity against the tested free radicals, with varying degrees of effectiveness.

Notably, the dichloromethane (DCM) extract exhibited the highest scavenging potential, with IC_50_ values ranging from 0.07 to 0.35 mg/mL, comparable to standard antioxidants (IC_50_: 0.07–0.43 mg/mL) against ABTS, hydroxyl (OH^•^), and nitric oxide (NO^•^) radicals.

**Figure 3:**
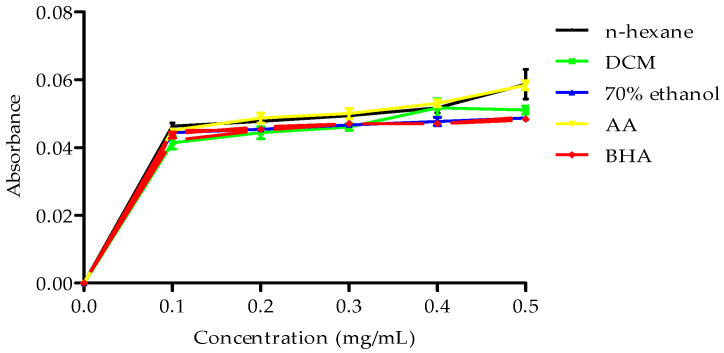
Reducing power of *B. bowkeri* crude extracts. Data are expressed as mean ± standard deviation (SD) (n = 3). Statistical significance was determined using one-way analysis of variance (ANOVA) followed by Dennett post hoc test, with *p* ≤ 0.05 considered to be statistically significant.

However, all the extracts showed poor metal iron chelating activity. Conversely, the extracts demonstrated reduction potential, with the n-hexane extract exhibiting higher reduction potential than the butylated hydroxyanisole (BHA) standard, as illustrated in Figure 3.

### 2.6. Inhibitory Effect of the Extracts on Enzyme Activity

#### 2.6.1. The Inhibitory Effects of n-Hexane

**Figure 4:**
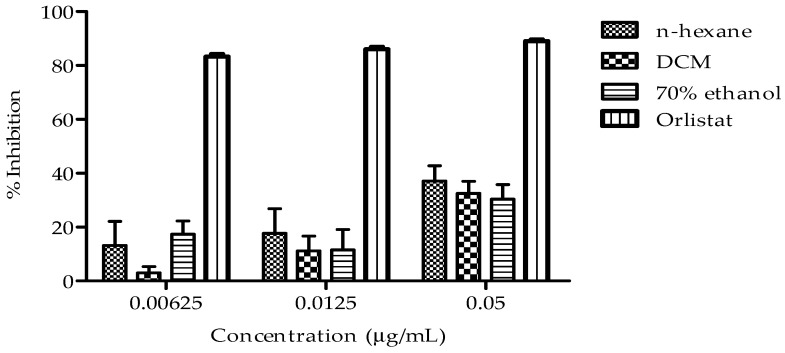
Presents the cholesterol esterase inhibitory activity of *B. bowkeri* extracts. Data are presented as mean ± standard deviation (SD) (n = 3). Statistical significance was determined by one-way analysis of variance (ANOVA) followed by Dennett post hoc test comparison among the groups, with *p* ≤ 0.05 considered to be statistically significant.

The effect of n-hexane, DCM, and 70% ethanol extracts on cholesterol esterase activity at three concentrations (0.00625, 0.0125, and 0.05 µg/mL) are shown in Figure 4. Notably, the n-hexane extract exhibited the most consistent and potent inhibitory effect on cholesterol esterase activity, with increasing inhibition observed across the tested concentrations when compared to other extracts.

#### 2.6.2. Effect of *B. bowkeri* Crude Extract on Pancreatic Lipase Activity

**Figure 5:**
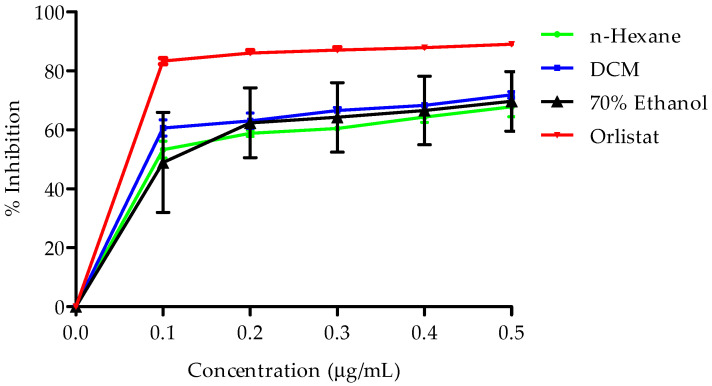
Presents pancreatic lipase inhibitory activity of *B. bowkeri* extracts. Data are expressed as mean ± standard deviation (SD). (n = 3.) Statistical significance was determined using one-way analysis of variance (ANOVA) followed by Dennett post hoc test, with *p* ≤ 0.05 considered to be statistically significant.

The inhibitory effects of the three extracts on pancreatic lipase activity were evaluated at various concentrations, and the results are illustrated in Figure 5. The extracts exhibited a dose-dependent inhibition of pancreatic lipase activity, with the following order of potency: DCM extract > n-hexane extract > 70% ethanol extract.

#### 2.6.3. Inhibition of the Extracts on HMG-CoA Reductase Activity

**Figure 6:**
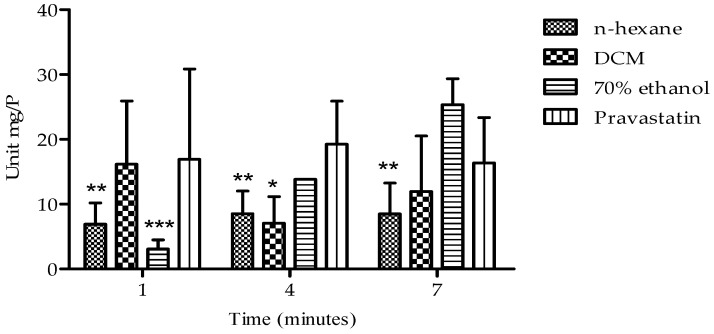
Presents HMG-CoA reductase inhibitory activity of *B. bowkeri* extracts. P = protein. Data are presented as mean ± standard deviation (SD) of three independent experiments (n = 3). Statistical significance was determined by one-way analysis of variance (ANOVA) followed by Tukey’s post hoc test. * *p* ≤ 0.05, ** *p* ≤ 0.01, and *** *p* ≤ 0.001 compared to the control group, pravastatin.

The effects of the extracts on HMG-CoA reductase activity are presented in Figure 6. All the extracts exhibited a time-dependent inhibitory effect on enzyme activity over a period of 7 min. Notably, the n-hexane extract demonstrated a significant and sustained inhibitory effect on HMG-CoA reductase activity between 4 and 7 min, with enzyme activity ranging from 8.51 ± 3.51 to 8.48 ± 4.76 Units/mgP. This inhibitory effect surpassed that of the standard pravastatin (19.26 ± 6.63 to 16.36 ± 7.01 Units/mgP).

### 2.7. The Effect of the Extracts on Bile Acid Binding

**Figure 7:**
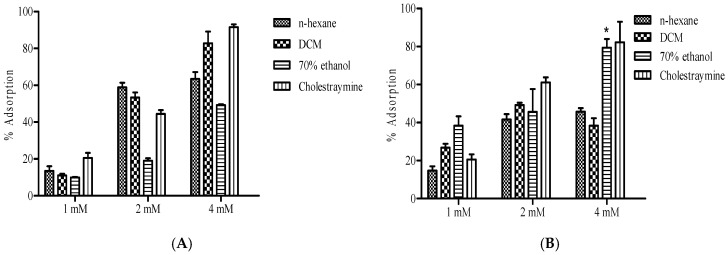
Presents the bile acid binding capacity of *B. bowkeri* extracts against (**A**) cholic acid and (**B**) deoxycholic acid. Data are presented as mean ± standard deviation (SD) of three independent experiments (n = 3). Statistical significance was determined by one-way analysis of variance (ANOVA) followed by Dennett post hoc test, with *p* ≤ 0.05 considered to be statistically significant. * *p* ≤ 0.01 compared to n-hexane and DCM extracts.

The bile acid binding capacity of *B. bowkeri* extracts was evaluated against cholic acid and deoxycholic acid. The results, presented in Figure 7, show the percentage absorption of residual bile acids. A concentration-dependent affinity for both cholic acid and deoxycholic acid was observed across all the extracts.

Notably, the extracts exhibited an increase in adsorption as the concentration increased against cholic and deoxycholic acid, comparable to the positive control cholestyramine.

### 2.8. Molecular Docking Analysis

**Figure 8:**
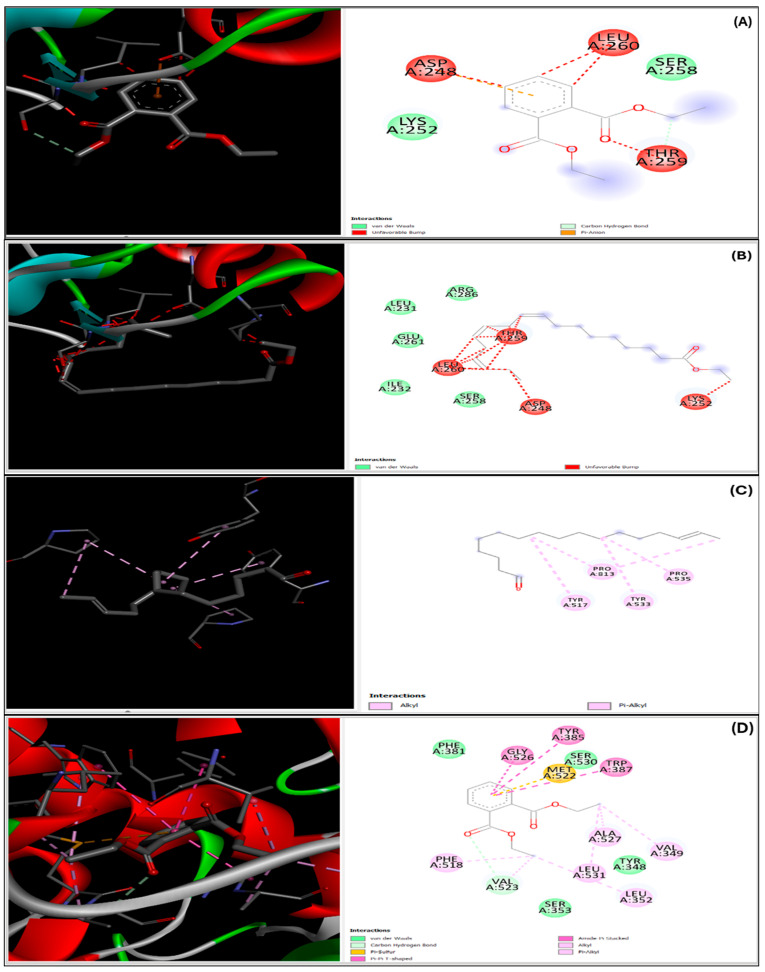

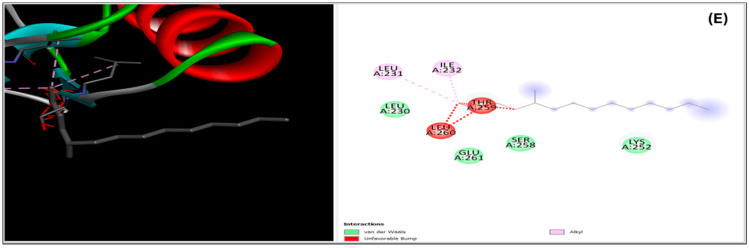
Presents the 2D and 3D representations of ligand–enzyme interactions, illustrating the binding between specific amino acids and complexes. The interactions are depicted for Diethyl Phthalate with 3FAK (**A**) and 4OTJ (**D**), ethyl ester (Z,Z) with 3FAK (**B**) and 4OTJ (**E**), and Diethyl Phthalate with 1HW9 (**C**).

The docking scores presented in Table 3 represent the binding affinities of primary compounds to target proteins accompanied by enzyme interaction binding between specific amino acids and complexes as shown in Figure 8, as well as standards, expressed in kilocalories per mole (kcal/mol). Interestingly, Diethyl Phthalate (DEP) demonstrated significant binding affinity to hormone-sensitive lipase (HSL) and cyclooxygenase (COX), with docking scores of −5.9 kcal/mol and −5.5 kcal/mol, respectively. These scores indicate a high binding affinity of DEP to HSL and COX. Moreover, the comparable docking scores of DEP and simvastatin indicate similar binding affinities of both compounds to the target proteins. Furthermore, the docking score of −7.0 kcal/mol for COX indicates the highest binding affinity of DEP among the evaluated proteins.

## 3. Discussion

The global health burden of elevated cholesterol levels and the associated complications persist, underscoring the urgent need for effective management and treatment strategies for hypercholesterolemia [17,18]. Although pharmaceutical treatments are available, their efficacy is often hindered by adverse effects, highlighting the need for alternative approaches. Plants have been traditionally utilized to alleviate various ailments, including hypercholesterolemia [19]. This study explores the anti-hypercholesterolemic potential of *B. bowkeri* crude extracts, with the aim of identifying novel, naturally derived therapeutic agents.

The results obtained in this study demonstrate that *B. bowkeri* bark extracts possess anti-hypercholesterolemic properties, as reflected by their inhibitory effect on 3-hydroxy-3-methylglutaryl-coenzyme A (HMG-CoA) reductase activity (Figure 6). Notably, HMG-CoA reductase is the rate-limiting enzyme in the hepatic biosynthesis of cholesterol, catalyzing the conversion of HMG-CoA to mevalonate. This enzyme plays a pivotal role in regulating the activation of sterol regulatory element-binding protein-2 (SREBP-2) and modulating the expression of low-density lipoprotein (LDL) receptors, ultimately leading to reduced cholesterol production and altered lipid profiles [20,21].

Numerous studies have reported that HMG-CoA reductase inhibitors are a prevailing and effective strategy for managing hypercholesterolemia [22,23]. The inhibitory effect of the extracts on HMG-CoA reductase activity (Figure 2B) may be attributed to the presence of flavonoids, which have been reported to inhibit HMG-CoA reductase enzyme activity, resulting in decreased mevalonate production. This mechanism is further supported by the antioxidant properties of flavonoids, which can donate hydrogen ions (H^+^) and prevent low-density lipoprotein (LDL) oxidation [24]. The potential benefits of flavonoids in managing hypercholesterolemia and preventing atherosclerosis are multifaceted, including enhanced antioxidant defenses, support for cardiovascular health, and regulation of cholesterol levels [25]. Therefore, flavonoids may be considered for dietary interventions, adjunct therapy to statin treatment, or natural product-based cardiovascular health supplements, highlighting their potential in promoting overall cardiovascular well-being [26].

During digestion, bile acids facilitate the emulsification of intestinal dietary fats into smaller particles, thereby enhancing their accessibility to enzymatic hydrolysis. Concomitantly, cholesterol esterase and pancreatic lipase catalyze the hydrolysis of dietary triglycerides into free fatty acids and glycerol, promoting efficient fat absorption [27]. Notably, inhibiting the activity of triglyceride-hydrolyzing enzymes, such as cholesterol esterase and pancreatic lipase, has emerged as a viable strategy for managing hypercholesterolemia and mitigating its associated complications [28]. This study provides evidence that *B. bowkeri* crude extracts exhibit significant inhibitory effects on cholesterol esterase (Figure 4) and pancreatic lipase (Figure 5) activities, supporting its anti-hypercholesterolemic properties. The inhibition of these enzymes is crucial in the management of hypercholesterolemia, as they play pivotal roles in lipid digestion and absorption [29]. Specifically, the inhibition of cholesterol esterase has been shown to reduce cholesterol absorption, whereas the inhibition of pancreatic lipase decreases triglyceride hydrolysis [30]. These findings are consistent with previous studies on plant extracts exhibiting anti-hypercholesterolemic effects through enzyme inhibition [31]. The observed inhibitory activities of *B. bowkeri* crude extracts on these enzymes suggest potential therapeutic applications in the management of hypercholesterolemia and related cardiovascular disorders.

The findings of this study are further substantiated by the extracts’ higher affinity for both primary (cholic acid) and secondary (deoxycholic acid) bile acids, which underscores their anti-hypercholesterolemic properties. Recent advances in understanding the biochemical mechanisms governing bile acid emulsification and cholesterol absorption have elucidated the complex interplay between bile acid biosynthesis, regulation, and lipid digestion [32,33]. Specifically, research has investigated the feedback regulation of cholesterol 7α-hydroxylase by sterols, providing insights into bile acid biosynthesis and regulation [34]. Furthermore, molecular studies on bile salt behavior at interfaces have enhanced our understanding of lipid digestion and absorption [35], while advances in producing analytical standards of bile acid glucuronides have enabled accurate quantification in biological samples [36].

Excessive blood cholesterol and its oxidation products contribute to the generation of reactive oxygen species (ROS), triggering oxidative damage to lipids, proteins, and nucleic acids and impairing physiological functions [37,38]. The interaction between ROS and nitric oxide (NO) produces peroxynitrite (ONOO-), a toxic compound that causes endothelial dysfunction, impaired vasodilation, increased vascular permeability, and pro-inflammatory responses [39,40]. Notably, plants with anti-hypercholesterolemic effects often exhibit strong antioxidant properties [41,42]. The crude extracts’ ability to scavenge free radicals (DPPH, ABTS, OH, nitric oxide, and metal iron chelating) and demonstrate reducing power (Table 2 and Figure 2) highlights their antioxidant potential, which may aid in combating oxidative stress-induced tissue damage. Recent evidence suggests that polyphenol-rich fruits, vegetables, and plants not only scavenge free radicals but also enhance endogenous antioxidant capacity [31]. The antioxidant activities of *B. bowkeri* crude extracts can be attributed to bioactive compounds detected via GC-MS analysis, including 9,12,15-Octadecatrienoic acid, ethyl ester (Z,Z), Diethyl Phthalate, E-15-Heptadecenal, Hexadecanoic acid, ethyl ester, and Tetradecane 5-methyl. Specifically, 9,12,15-Octadecatrienoic acid, ethyl ester (Z,Z) and Hexadecanoic acid, ethyl ester have reported antioxidant properties [43], while Diethyl Phthalate exhibits antioxidant activity by scavenging free radicals [44]. These findings suggest that the antioxidant potential of *B. bowkeri* extracts may be partially attributed to these bioactive compounds, contributing to their therapeutic applications.

Furthermore, the computational technique which predicts how ligand binds to target proteins was employed to further support the possible mechanism through which the extracts exhibited their anti-hypercholesterolemic effects. Thus, the molecular docking evaluation of *B. bowkeri* crude extract revealed potential inhibitory activity against key enzymes implicated in hypercholesterolemia and inflammation, including cyclooxygenase (COX), hormone-sensitive lipase (HSL), and 3-hydroxy-3-methylglutaryl-coenzyme A (HMG-CoA) reductase. Notably, compounds present in the extract, such as Diethyl Phthalate, Tetradecane 5-methyl, E-15-Heptadecenal, and 9,12,15-Octadecatrienoic acid ethyl ester (Z,Z), demonstrated promising binding affinity to these enzymes. Hypercholesterolemia is associated with increased activity of lipoxygenase, phospholipase A2, and COX, leading to excessive production of pro-inflammatory leukotrienes and prostaglandins via arachidonic acid metabolism [45,46]. This contributes to chronic inflammation and oxidative stress [47]. By inhibiting these enzymes, the bioactive compounds in *B. bowkeri* may mitigate chronic inflammatory responses and associated pathological conditions [48], positioning them as potential therapeutic agents for managing hypercholesterolemia and inflammation.

## 4. Materials and Methodology

### 4.1. Materials

#### 4.1.1. Chemical Reagents

All the chemicals and reagents utilized in this study were procured from Sigma-Aldrich Chemical Co., St. Louis, MO, USA, and were of analytical grade unless explicitly mentioned otherwise.

#### 4.1.2. Collection and Extraction of *Bauhinia bowkeri*

Fresh bark of *B. bowkeri* was collected from Pongola, in KwaZulu-Natal, South Africa (27.3798° S, 31.6219° E). The plant was identified and authenticated by Prof N. Ntuli from the Botany Department, University of Zululand. A voucher specimen (Specimen No. V12) was deposited at the herbarium of the Department of Botany, University of Zululand, South Africa. The collected plant material was air-dried at room temperature and ground into fine powder using an IKA-Werke grinder (Polychem) with a 2 mm mesh sieve. The powdered material was sequentially extracted (1:5 *w*/*v*) using solvents of increasing polarity: n-hexane, dichloromethane (DCM), and 70% ethanol. Each extraction was carried out for 48 h under continuous agitation in a Labcon orbital shaker (180 rpm) at 25 °C, with the solvent refreshed every 24 h. The total extraction time was 144 h. The extracts were filtered through Whatman No. 1 filter paper and concentrated under reduced pressure using a Heidolph rotary evaporator (Heidolph Laborota 4000, Schwabach, Germany) at 40 °C. The resulting crude extracts were stored in sterile brown bottles at 4 °C until further use.

### 4.2. Methodology

#### 4.2.1. Phytochemical Profiling of *B. bowkeri* Extracts by FT-IR

The FT-IR spectra of the plant extracts were recorded using a Perkin Elmer Spectrum Two FT-IR spectrophotometer equipped with a universal attenuated total reflectance (UATR) accessory. The spectral data were collected over the wavenumber range 370–4000 cm^−1^. The functional groups were identified by comparing the observed peak frequencies with a standard IR spectroscopy data table [49].

#### 4.2.2. Phytochemical Profiling of *B. bowkeri* Extracts by GC-MS

The phytochemical composition of the *B. bowkeri* crude extracts was analyzed using GC-MS (Agilent 6890 is an equipment of Agilent Technologies 2850 Centerville Rd, Wilmington, DE 19808, Delaware, USA). The system was equipped with a fused silica capillary column coated with DB-5. The ionization energy was set to 70 eV, and the injector temperature was maintained at 250 °C. The oven temperature was programmed to increase gradually from 40 °C to 280 °C at a rate of 3 °C per minute. Helium was used as the carrier gas. The compounds were identified by direct comparison of the mass spectrum of the analyte at a particular retention time to that of reference standards found in the 2011 National Institute of Standards and Technology (NIST) library.

#### 4.2.3. Quantification of Total Phenolic Content (TPC)

The TPC of the *B. bowkeri* extracts was determined using the Folin–Ciocalteu method, following the procedure outlined by Kähkönen et al. [50]. Briefly, the extracts solution (0.5 mg/mL) were mixed with Folin−Ciocalteu reagent (diluted 1:10 *v*/*v*) and 7.5% sodium carbonate solution. The mixture was incubated in the darkness at 25 °C for 30 min. The absorbance was measured at 765 nm using a Biotek Synergy HT microplate reader. The TPC was quantified as gallic acid equivalent (GAE) by comparing the absorbance values to a standard calibration curve generated using the gallic acid standard.

#### 4.2.4. Quantification of Total Flavonoid Content (TFC)

The TFC of the *B. bowkeri* extracts was determined following the protocol described by Ordonez et al. [51]. Briefly, extracts concentration of (0.5 mg/mL) were mixed with 2% alcoholic aluminium chloride solution and incubated at room temperature for 1 h. The absorbance was then measured at 420 nm using a Biotek Synergy HT microplate reader (Synergy HT, BioTek Instrument, Inc., Winooski, VT, USA) with a blank containing alcoholic aluminium chloride serving as the reference. The TFC was quantified and expressed as quercetin equivalent (QE) using a calibration curve generated with quercetin.

#### 4.2.5. In Vitro Antioxidant Assays

The antioxidant potential of the *B. bowkeri* crude extracts was evaluated against various inorganic free radicals, including 1,1-diphenyl-2-picryl hydrazyl (DPPH), 2, 2-azinobis 3-ethylbenzothiazoline-6-sulfonate (ABTS), hydroxyl radicals (OH^•^), and nitric oxide (NO). Additionally, the metal iron chelating activity and reducing power of the extracts were also evaluated. Ascorbic acid (AA) and butylated hydroxylanisole (BHA) were used as the reference standards, unless otherwise specified. The free radical scavenging activity of each extract was calculated and expressed as a percentage of inhibition.

Scavenging activity (%) = ((A_c_ − A_t_)/A_c_ × 100)

where

A_c_ = the absorbance of the control sample;
A_t_ = the absorbance of the sample in the presence of the tested extract.
The absorbances were measured with a microplate reader (Synergy HT, BioTek Instrument, Inc., Winooski, VT, USA).

The ABTS radical scavenging activity of *B. bowkeri* was evaluated following the method described by Re et al. [52]. ABTS radicals were generated by incubating 7 mM ABTS with 2.45 mM potassium persulfate for 16 h in the dark, followed by dilution with ethanol (1:60). The ABTS solution (1 mL) was mixed with 1 mL plant extract (0–0.5 mg/mL), and the absorbance was measured at 734 nm after 6 min using a Biotek Synergy HT microplate reader.

The DPPH radical scavenging activity was evaluated using the method described by Williams [53]. The extracts (0–0.5 mg/mL) were mixed with 0.2 mg/mL DPPH solution in a 1:1 ratio and incubated in the dark for 60 min. The absorbance was measured at 517 nm using a Biotek Synergy HT microplate reader. Ethanol and ascorbic acid were used as the controls. The percentage of inhibition was calculated to determine the antioxidant activity.

The hydroxyl radical (OH^•^) scavenging activity of the *B. bowkeri* crude extracts was determined following the method described by Halliwell and Gutteridge [54]. The reaction mixture contained 10 mM FeSO_4_·H_2_O, extract concentration (0–0.5 mg/mL), 10 mM H_2_O_2_, 10 mM EDTA, and 100 mM phosphate buffer (pH 7.4). The mixture was incubated at 37 °C for 2 h followed by the addition of 2.8% trichloroacetic acid (TCA) and 1% thiobarbituric acid (TBA). The solution was boiled for 10 min, cooled on ice, and the absorbance measured at 520 nm using a Biotek Synergy HT microplate reader.

The metal iron chelation ability of *B. bowkeri* crude extracts was evaluated according to the method described by Decker et al. [55]. The reaction mixture contained 5 mM ferrozine, 2 mM FeCl_2_, extract concentrations (0–0.5 mg/mL), and deionized water. The mixture was incubated at 25 °C for 10 min. Post-incubation, the absorbance was measured at 562 nm using a UV-Vis spectrophotometer Biotek Synergy HT microplate reader. Ethylenediaminetetraacetic acid (EDTA) was used as the reference standard.

The reducing power of the *B. bowkeri* crude extracts was evaluated following the method described by Oyaizu [56]. Briefly, 2.5 mL of extract solution (0–0.5 mg/mL) was mixed with 1% potassium ferricyanide and 200 mM phosphate buffer (pH 6.6) in a test tube. The mixture was incubated at 50 °C for 20 min, followed by centrifugation at 119× *g* for 10 min. The supernatant was then mixed with 0.1% ferric chloride. The reduction potential was determined by measuring the absorbance at 700 nm using a Biotek Synergy HT microplate reader.

#### 4.2.6. In Vitro Enzyme Inhibitory Assays

##### Inhibitory Effect of the Extract on Cholesterol Esterase Activity

The cholesterol esterase inhibitory activity of the crude extract was evaluated using a modified method described by Mulvihill et al. [25]. The reaction mixture contained 50 μL of 100 mM sodium phosphate-buffered saline (pH 7.0), 50 μL of 5.16 mM taurocholic acid, 50 μL of 100 μg/mL cholesterol esterase, 50 μL crude extracts (0–0.5 µg/mL), and 75 μL of 0.2 mM para-nitrophenol-butyrate (p-NPB) in 6% acetonitrile. Orlistat was used as the reference standard. The mixture was incubated at 25 °C for 10 min before adding the substrate. The reaction was further incubated for 5 min at 25 °C, and the absorbance was measured at 405 nm using a Biotek Synergy HT microplate reader. The percentage of enzyme inhibition was calculated using the following equation:

Inhibitory activity (%) = ((A_c_ − A_t_)/A_c_ × 100)

where

A_c_ = the absorbance of the control sample;
A_t_ = the absorbance of the sample in the presence of the tested extract.
The absorbances were measured with a microplate reader (Synergy HT, BioTek Instrument, Inc., Winooski, VT, USA).

##### Inhibitory Effect of the Extract on Pancreatic Lipase Activity

The pancreatic lipase inhibitory activity of the *B. bowkeri* crude extracts was evaluated using the method described by Slanc et al. [57]. The reaction mixture contained 125 μL of Tris-HCl buffer (75 mM, pH 8), 75 μL of extract at varying concentrations (0–1.5 μg/mL), and 50 μL of pancreatic lipase (10 μg/mL). The mixture was incubated at 37 °C for 15 min before initiating the reaction by adding 25 μL of 3.3 mM 4-nitrophenyl palmitate. The reaction mixture was further incubated at 37 °C for 10 min. A blank solution containing ethanol and water (1:1 *v*/*v*) was used as a control, while orlistat served as the reference inhibitor. The enzyme activity was determined by measuring the release of p-nitrophenol at 405 nm using a Biotek Synergy HT microplate reader. The percentage of inhibition was calculated using the following equation.

##### Inhibitory Effect of the Extract on HMG-CoA Reductase Activity

The inhibitory effect of the *B. bowkeri* extracts on HMG-CoA reductase activity was evaluated following the method described by Friesen and Rodwell [22]. Extract solutions at varying concentrations (0–1.5 µg/mL) were prepared, centrifuged, and filtered before being mixed with 364 µL of assay buffer, 24 µL of HMG-CoA substrate, 8 µL of NADPH, and 2 µL of enzyme. A mixture of 366 µL of 1× assay buffer, 24 µL of HMG-CoA substrate, 8 µL of NADPH, and 2 µL of HMG-CoA reductase substrate was made. The reaction was incubated at 37 °C, and the absorbance was measured at 340 nm using a Biotek Synergy HT microplate reader at 1 min intervals for 10 min. Simvastatin was used as a reference inhibitor.

$$\mathrm{Unit}/\mathrm{mP}=\frac{∆A340/minsample-∆A340/minBlank\;}{12.44\times V\times 0.6\times LP}\times TV$$

where

12.44 represents the 2 NADPH consumed;
TV = the total volume of the reaction (mL);
*V* = the volume of the enzyme used;
0.6 = the enzyme concentration (mg-protein, mgP);
*LP* = the light path (cm).

#### 4.2.7. The Effect of Extracts on Bile Acid

The bile acid binding activity of the *B. bowkeri* crude extract was evaluated following the method described by Matsumoto et al. [58]. Briefly, bile acid solutions at varying concentrations (0–1.5 mM) (cholic acid (CA) and deoxycholic acid (DCA)) were prepared in phosphate-buffered saline (0.1 M, pH 7.4). These bile acids were then incubated with 1% (*w/v*) of the extract for 30 min at 37 °C with intermittent shaking. Following incubation, the mixture was centrifuged at 20,817× *g* for 5 min, and the supernatant was analyzed for the residual bile acid concentration using a bile acid assay kit. Cholestyramine served as a positive control. The bile acid levels were calculated using the following equation:

(%) Inhibitory effect = ((A_c_ − A_t_)/A_c_ × 100)

#### 4.2.8. In Silico Studies

In silico studies were conducted to assess the binding affinities of GC-MS-identified compounds from *B. bowkeri* crude extracts with HMG-CoA reductase, secretory phospholipase A_2_ (sPLA_2_), and cyclooxygenase (COX). The 2D structures of these proteins were retrieved from the Protein Data Bank (PDB). Molecular docking was performed using Auto-Dock Tools (version 1.5.4), where appropriate grid box sizes were determined for potential binding sites, and Gasteiger charges were assigned prior to docking. The binding affinities were subsequently analyzed using a software UCSF Chimera version 1.17.1 (UCSF, San Fransisco, CA, USA).

#### 4.2.9. Statistical Analysis

All the data were expressed as the mean ± standard deviation (M ± SD). The data analysis, as well as the IC_50_ values, was calculated using a software GraphPad Prism version 9.3.1. One-way ANOVA followed by a Dennett post hoc test was used to compare the results to determine the variance among the samples; the *p* ≤ 0.05 value was statistically significant.

## 5. Conclusions

The present study provides compelling evidence that *B. bowkeri* extracts possess significant anti-hypercholesterolemic properties, highlighting their potential as a therapeutic agent in the management and treatment of hypercholesterolemia. The observed anti-hypercholesterolemic effects of the extracts can be attributed to their multifaceted mechanisms of action, including their antioxidant properties and enzyme inhibitory activities against cholesterol esterase, pancreatic lipase, and HMG-CoA reductase, as well as their bile acid binding capabilities. Furthermore, the molecular docking simulations also provided additional support for the anti-hypercholesterolemic effects of *B. bowkeri* extracts, indicating the interaction of the crude extracts with key enzymes involved in cholesterol metabolism. These findings suggest that *B. bowkeri* extracts may serve as a potential therapeutic agent in the treatment and management of hypercholesterolemia, providing a novel and naturally derived approach for mitigating this prevalent cardiovascular disease.

## Figures and Tables

**Table 1:** Major compounds identified in different extracts of *B. bowkeri* by GC-MS analysis. (Average height percentage and retention time.)

Compound Names	RT	% Area	Molecular Formula	Molecular Mass
9,12,15-Octadecatrienoic acid, ethyl ester	23.06	10.61	C_20_H_36_O_2_	308
Diethyl Phthalate	13.44	27.60	C_12_H_14_O_4_	222
E-15-Heptadecenal	18.49	18.00	C_18_H_36_O	284
Hexadecanoic acid, ethyl ester	15.72	7.10	C_17_H_32_O	252
9,12,15-Octadecatrienoic acid, ethyl ester, (Z,Z)	23.29	6.10	C_20_H_34_O	306
Tetradecane 5-methyl	25.38	10.36	C_15_H	212
Octadecane, 5-methyl	25.42	10.62	C_19_H	268

**Table 2:** IC_50_ values of different extracts of *B. bowkeri* crude extract (mg/mL).

Extracts IC_50_ (mg/mL)	DPPH	ABTS	OH^•^	NO^•^	Metal Ion Chelating
n-hexane	-	0.08 ± 0.01	0.25 ± 0.06	0.41 ± 0.04	-
DCM	-	0.07 ± 0.00	0.16 ± 0.02	0.35 ± 0.03	-
70% ethanol	0.38 ± 0.01	0.07 ± 0.01	0.13 ± 0.02	0.32 ± 0.05	-
AA	0.28 ± 0.01	0.20 ± 0.13	0.09 ± 0.00	0.30 ± 0.17	3.97 ± 11.31
BHA	0.18 ± 0.17	0.07 ± 0.00	0.25 ± 0.02	0.34 ± 0.07	3.74 ± 11.93

Antioxidant activities of *B. bowkeri* crude extracts (n-hexane, DCM, and 70% ethanol) AA: ascorbic acid and BHA: butylated hydroxyanisole. Data are expressed as mean ± standard deviation (SD) (n = 3). Statistical significance was determined using one-way analysis of variance (ANOVA) followed by Dennett post hoc test. *p* ≤ 0.05 indicates significant differences among the groups.

**Table 3:** Docking scores of tested compounds against selected target proteins from *B. bowkeri*.

Compound Names	3 FAK Hormone-Sensitive Lipase (Kcal/mol)	1 HW9 HMG CoA Reductase (Kcal/mol)	4 OTJ Cyclooxygenase (Kcal/mol)	Simvastatin (Kcal/mol)	PUBCHEM ID
Diethyl Phthalate	−5.9	−5.4	−7.0	−5.5	6781
Tetradecane 5-methyl	−4.9	−3.9	−4.5	−4.4	98,976
E-15-Heptadecenal	−4.1	−4.1	−4.7	−4.2	5,363,097
9,12,15-Octadecatrienoic acid, ethyl ester (Z,Z)	−5.4	−4.6	−5.1	−4.6	5,363,097

## Data Availability

The supporting data will be available on request from the authors.
